# Editorial: Personalized therapies for monogenic diabetes

**DOI:** 10.3389/fgene.2024.1496367

**Published:** 2024-09-27

**Authors:** Maurizio Delvecchio, Ming Liu, Novella Rapini, Fabrizio Barbetti

**Affiliations:** ^1^ Department of Biotechnological and Applied Clinical Sciences, University of L’Aquila, L’Aquila, Italy; ^2^ Department of Endocrinology and Metabolism, Tianjin Medical University General Hospital, Tianjin, China; ^3^ Endocrinology and Diabetes Unit, Bambino Gesù Children’s Hospital, IRCCS, Scientific Institute for Research, Hospitalization and Health Care, Rome, Italy; ^4^ Monogenic Diabetes Clinic, Unit of Endocrinology and Diabetes, Bambino Gesù Children Hospital IRCCS, Rome, Italy

**Keywords:** monogenic diabetes, personalized medicine, MODY, neonatal diabetes mellitus, Rabson-Mendenhall syndrome, severe insulin resistance syndrome, gestational diabetes, Mendelian randomization

Monogenic diabetes is the best example of a precision medicine approach to the management of diabetes (Naylor et al., 2024). It has three main clinical forms: 1) Maturity Onset Diabetes of the Young (MODY), which is an autosomal dominant disorder, 2) Neonatal Diabetes Mellitus (NDM), which is usually an autosomal dominant disorder often caused by a *de novo* mutation or autosomal recessive, often syndromic and 3) the syndromes of Severe Insulin Resistance (SIRs), which can be dominant or recessive inherited disorders. MODY and NDM are caused by pathogenic variants in genes that affect pancreatic beta cell development, survival, and/or function, while SIRs are caused by pathogenic variants in genes that affect the insulin action causing high to massive hyperinsulinemia.

It is to note that practice consensus guidelines regarding MODY subtypes exist about GCK-MODY and HNF1A-MODY, since robust data are available in literature. Limited evidence from clinical trials is available about the other types of MODY/NDM and most of knowledge on this Research Topic are from case reports and case series.

Basically, mutations in GCK, HNF1A, HNF4A, and HNF1B genes account for more than 95% of MODY patients worldwide (Colclough et al., 2022; Saint-Martin et al., 2022). As carriers of GCK variants do not need any treatment, patients bearing either HNF1A or even HNF4A mutations can respond to oral hypoglycemic agents (OHA) of the class of sulfonylureas (SU) or other drugs different from insulin (Delvecchio et al., 2020). NDM is caused by mutations in more than 40 beta-cell genes, but two of them (KCNJ11 and ABCC8) account for about 50% of cases. These two genes are actionable, with most carriers reaching optimal metabolic control with SU treatment (Bowman et al., 2018; Bowman et al., 2021).

Finally, SIRs may respond to human recombinant IGF1, leptin, thiazolidinediones and sodium–glucose cotransporter two inhibitors (SGLT2is), depending on subtype.

This Research Topic aims to provide an updated view of treatment options for patients with monogenic diabetes. A special attention to mechanisms of disease and appropriate drug choice for any specific condition (precision medicine) has been paid, to inform the readers about new treatments and repurposing of old or even new therapies. Eleven papers were submitted for publication and four of them (36%; one mini-review, one case report, and two original reports) were accepted for publication.

Treatment of MODY during pregnancy is still a field of research, because it needs tailored care to the pregnant woman and the offspring. However, different mechanisms of disease and metabolic phenotypes among MODY subtypes dictate for diverse treatments and this applies to pregnant women with MODY. Unfortunately in many individuals this condition may remain undiagnosed or misdiagnosed until pregnancy. In their mini-review, Crowley et al. reviewed data about pregnancies and offspring health in MODY women and in pregnancies with paternally inherited MODY gene mutation. In the case of SU pre-pregnancy treatment, switch to insulin is suggested. Macrosomia occurs in more than half of the cases and thus fetal growth must be monitored and the treatment tailored on the basis of genetic and ultrasound findings. In these newborns, hypoglycemia may occur up to 48 h of life. On the other hand, in the case of GCK/MODY, the management is different. Available data show that fetal DNA testing combined with ultrasound is useful to choose the most appropriate treatment, and different treatments strategies have to be chose on the basis of these findings. Very interesting suggestions for appropriate management are provided.

The Italian group led by prof. Bonfanti (Foglino et al.) described the management of a young boy with the Rabson–Mendenhall syndrome treated with SGLT2is. This syndrome is characterized by SIR, very challenging to treat. They investigated the benefit of adding emplaglifozin to insulin treatment to improve blood glucose control. This approach was successful with HbA1c decrease from 8.5% to 7.1% after 10 months of SGLT2is. The treatment of this very rare syndrome is difficult and different strategies were attempted. This paper highlights the benefits of this new drug in adjunction to insulin therapy, to reduce hyperglycemia with blood glucose control improvement. The authors wisely suggest that this approach can be suggested in infants with SIRs but more evidence from other cases is mandatory to get robust conclusions about its actual benefit.

Congenital lipodystrophy is a rare disease which causes metabolic disease and lipoatrophic diabetes. Fat tissue can be completely or partially absent. Research for effective treatment is still ongoing. In their paper, Roumane et al. investigated the effects of a glucagon like peptide-1 receptor agonist (GLP1RA) in mice with generalized lipodystrophy. Twelve mice were treated with liraglutide, acutely before insulin assay and glucose tolerance evaluation or chronically before metabolic profile investigation and *ex vivo* studies. The results are encouraging, as liraglutide has been effective to improve insulin secretion and glucose tolerance. Hepatomegaly, steatosis and markers of liver fibrosis were significantly reduced as well. The authors concluded that liraglutide can be an effective treatment for individuals with congenital lipodystrophy.

Personalized treatment approach based on genetic findings is commonly named as precision medicine. Mendelian randomization is a method which uses measured variation in genes to investigate the causal effect of an exposure on an outcome. It is used to discover the epidemiology and pathogenesis of diseases or possible therapeutic targets. Through this method, Li et al. investigated potential treatment targets in type 1 and type 2 diabetes to provide new strategies for the treatment and to suggest a possible priority in drug development. They evaluated more than 4,000 druggable genes, identifying 14 potential druggable genes that could affect the therapeutic outcomes, 7 in type 1 diabetes and other 7 in type 2 diabetes. In the future, it is very likely that more research like this will be available, supporting clinicians in the treatment choice.
